# Web-Based, Algorithm-Guided Insulin Titration in Insulin-Treated Type 2 Diabetes: Pre-Post Intervention Study

**DOI:** 10.2196/68914

**Published:** 2025-02-07

**Authors:** Nishanth Thiagarajan, Hong Chang Tan, Suresh Rama Chandran, Phong Ching Lee, Yun Ann Chin, Wanling Zeng, Emily Tse Lin Ho, David Carmody, Su-Yen Goh, Yong Mong Bee

**Affiliations:** 1 Department of Endocrinology, Singapore General Hospital, 20 College Road, AcademiaSingapore, 169856, Singapore, 65 88900691

**Keywords:** diabetes, insulin, monitoring, technology, mobile, app, intervention

## Abstract

**Background:**

Self-monitoring of blood glucose (SMBG) using web-based diabetes management platforms has demonstrated promise in managing type 2 diabetes (T2D). However, the effectiveness of such systems incorporating algorithm-guided insulin titration has not been extensively studied in Asian populations.

**Objective:**

This study evaluates the efficacy and safety of the ALRT telehealth solution—a US Food and Drug Administration–cleared, web-based platform that integrates SMBG with algorithm-driven insulin dose adjustments—in improving glycemia in insulin-treated T2D.

**Methods:**

This 24-week, pre-post intervention study enrolled 25 adults with T2D (mean age 58.9, SD 7.0 y; n=14, 56% male) on twice-daily premixed insulin. Inclusion criteria included a baseline hemoglobin A_1c_ (HbA_1c_) level between 7.5% to 9.9% (58‐86 mmol/mol), a BMI ≤40 kg/m², and experience with SMBG. Participants uploaded twice-daily SMBG data weekly via a mobile app, which generated insulin titration recommendations based on a predefined algorithm. Physicians reviewed and approved the recommendations, which were then communicated back to participants via the app. The primary outcome was the change in HbA_1c_ level from baseline to 24 weeks. Secondary outcomes included changes in fasting plasma glucose, insulin dose, hypoglycemia incidence, and SMBG adherence.

**Results:**

Participants achieved significant reductions in HbA_1c_ level from 8.6% (70 mmol/mol) at baseline to 7.4% (57 mmol/mol) at 24 weeks (*P*<.001), with reductions of 0.8% and 0.4% in the first and second 12 weeks, respectively. Fasting plasma glucose decreased from 8.7 (SD 2.0) mmol/L to 7.1 (SD 1.4) mmol/L (*P*<.001). Mean total daily insulin dose increased modestly from 0.73 (SD 0.31) units/kg/day to 0.79 (SD 0.34) units/kg/day (*P*=.007). Participants demonstrated high adherence, completing 97.3% (327/336) of prescribed SMBG measurements. During the study, 48% (12/25) of participants experienced at least 1 hypoglycemia episode, predominantly mild hypoglycemia (85/96, 88.5%; glucose 3.0‐3.9 mmol/L). Hypoglycemia episodes increased from 24 during weeks 0‐12 to 72 during weeks 13‐24. There were no episodes of severe hypoglycemia requiring external assistance. BMI increased slightly from 29.0 (SD 3.6) kg/m² to 29.5 (SD 3.6) kg/m² (*P*=.03), reflecting a modest weight gain associated with improved glycemia.

**Conclusions:**

In conclusion, patients with insulin-treated T2D initiated on a web-based glucose monitoring system with algorithm-guided dosing recommendations showed significant improvement in glycemic control compared to baseline. High adherence rates underscore the feasibility of integrating algorithm-guided insulin titration into routine care. While hypoglycemia incidence rose slightly, episodes were predominantly mild, and no severe events occurred. This intervention shows promise for broader adoption in T2D management, particularly in resource-constrained settings.

## Introduction

The global prevalence of diabetes mellitus has risen dramatically, with projections estimating that over 1.3 billion people will be affected by 2050 [1]. This chronic disease profoundly impacts both quality of life [2] and health care expenditure [3], and has driven significant efforts toward early detection and treatment.

Effective glycemic control remains the corner stone of diabetes management, correlating with improved long-term outcomes [4-6]. Despite rapid advances in pharmacotherapy, up to two-thirds of patients fail to achieve their glycemic targets [7]. Self-monitoring of blood glucose (SMBG) is a pivotal component of diabetes management [8], enhancing patient engagement and glycemic control [9]. The benefits of SMBG are most pronounced [10-12] when a structured approach is taken. Ideally, blood glucose readings should be collected at specified intervals and returned to health care providers for interpretation. In this regard, a safe and consistent dose adjustment regimen will be needed if any treatment modification is required. Finally, patients need to be promptly informed of the necessary changes. Thus, structured SMBG’s effectiveness is contingent on regular, frequent physician reviews and adjustments of therapy [13,14].

However, having frequent in-person clinic visits to review SMBG and adjust insulin doses is impractical, given the massive patient load and long waiting time of many diabetes clinics. Furthermore, a vast amount of SMBG data needs to be reviewed and interpreted by the physician during the limited allocated clinic time. This process is laborious and time-consuming, yet decision-making for insulin dose adjustments needs to be rapid. Providers with less clinical experience will find this especially challenging. Consequently, the SMBG data are often not fully capitalized to improve a patient’s diabetes management.

Several web-based programs and mobile apps have been developed to overcome some of the abovementioned challenges. Instead of manual recording of SMBG and then providing feedback to their provider in person, these programs allow patients to record and transmit SMBG data directly via the app, reducing the frequency of face-to-face visits [15-17]. In an earlier study, we demonstrated the ability of a web-based blood glucose monitoring system to optimize insulin dosages for patients receiving a basal-plus or basal-bolus insulin regimen. Importantly, glycemic control improved without increasing hypoglycemia rates [18]. However, although this approach reduced the need for face-to-face visits for insulin titration, physicians still need to review a large amount of SMBG data before recommending any insulin dose adjustment. In addition, insulin doses are adjusted based on the physician’s discretion and experience. Such an approach requires extensive workforce resources and is unlikely to solve the real-world problem of delays in treatment optimization. One potential solution is to use an automated insulin dose adjustment system that could interpret SMBG data and provide dose adjustment recommendations based on a preprogrammed algorithm. Studies have demonstrated the superiority of such systems in improving glycemic control compared to physician-dependent adjustments alone [19]. However, such programs have not been studied in an Asian cohort.

We thus designed the Glucose Monitoring and Intervention in Insulin-treated Type 2 Diabetes Patients (GEMINI-T2D) study to examine the efficacy and safety of a web-based platform that incorporates monitoring and insulin titration recommendations based on a built-in algorithm in patients treated with insulin.

## Methods

### Study Design

The GEMINI-T2D study was a prospective, single-site, single-group, pre-post interventional study.

### Study Participants

All participants were recruited from the Singapore General Hospital’s Diabetes & Metabolism Centre from September 2020 to May 2022. Those deemed potentially eligible were contacted by phone, by email, or during scheduled visits to the Diabetes & Metabolism Centre. Those who expressed interest in participating were given the informed consent form and invited to participate. Final eligibility of those who consented to participate was based on satisfactory completion of the screening process, demonstrating full eligibility based on inclusion and exclusion criteria.

### Inclusion Criteria

We recruited adults (aged ≥21 y) with type 2 diabetes (T2D) of more than 6-month duration on twice-daily premixed insulin regimen for ≥3 months. The total daily dose (TDD) of insulin must be <1 unit/kg, hemoglobin A_1c_ (HbA_1c_) level must be between 7.5% (58 mmol/mol) to 9.9% (86 mmol/mol), and BMI must be ≤40 kg/m². We chose to study patients on premixed insulin as it represents a common and preferred insulin formulation in Asia [20,21]. Participants were required to have experience performing SMBG and own a compatible smartphone capable of weekly data uploads via the study app.

### Exclusion Criteria

We excluded individuals with hypoglycemia unawareness (Gold score ≥4) to limit the risk of severe hypoglycemia during treatment. Pregnant or breastfeeding women, as well as those with severe renal impairment (estimated glomerular filtration rate <30 mL/min/1.73 m²), hemoglobinopathies, systemic corticosteroid use, and medical disease with a life expectancy of less than 1 year, were excluded.

### Intervention

Eligible participants were given a glucometer and instructed to perform SMBG twice daily. They were trained to use the ALRT system—a US Food and Drug Administration–cleared diabetes management system—and asked to upload their SMBG data to the ALRT app weekly for the next 24 weeks. Participants were also educated on hypoglycemia recognition and management, and instructed to perform SMBG when experiencing symptoms suggestive of hypoglycemia.

### Insulin Dose Adjustment

The ALRT system analyzed uploaded SMBG data every 7 days. When capillary blood glucose readings consistently fell outside the predefined range of 4‐8 mmol/L, a predefined algorithm adjusted the prebreakfast and/or predinner insulin doses, implementing an increment or decrement of up to 15% to 20% as necessary (details of the algorithm are provided in Multimedia Appendix 1). The treating physician was notified via a web-based user interface with the recommended insulin dose adjustments and was required to accept or decline the recommendation. Subsequently, the adjusted insulin doses were communicated to the participants via the ALRT app every 7 days, or more frequently in the event of hypoglycemia. Participants were required to acknowledge the recommended dose adjustments via the app.

### Baseline and Follow-Up Assessments

Medical history, vital signs, body measurements, and laboratory measurements were collected at baseline. HbA_1c_ level and fasting plasma glucose (FPG) were measured at baseline, week 12, and week 24.

### Outcome Measures

The primary outcome measure was the change in HbA_1c_ level at the end of 24 weeks relative to baseline. Secondary outcome measures included the incidence of hypoglycemia, defined as the number of episodes of hypoglycemia per participant, and the adherence to the prescribed SMBG regimen. Episodes of hypoglycemia were classified as follows:

- Level 1: Glucose value of 3.0‐3.9 mmol/L
- Level 2: Glucose value of <3.0 mmol/L
- Level 3: Severe hypoglycemia causing altered mentation requiring external assistance for recovery

Adherence to the SMBG regimen was defined as the proportion of the prescribed twice-daily SMBG that the participant successfully completed and uploaded to the ALRT platform. The incidence of hypoglycemia was defined as the number of episodes of hypoglycemia that occurred during each 12-week period of the study.

### Statistical Analysis

Continuous data were presented as mean (SD) and categorical data were presented as numbers (percentages). We compared the variables at baseline, week 12, and week 24, using repeated-measures ANOVA for normally distributed variables with post hoc Tukey method, and Friedman ANOVA for nonnormally distributed variables with post hoc Wilcoxon signed-rank tests with Bonferroni correction. Statistical analyses were performed using R (version 4.1.1; R Foundation for Statistical Computing).

### Ethical Considerations

The SingHealth Institutional Review Board reviewed and approved the study (reference 2019/2874). Informed consent was obtained from all participants, and this was inclusive of secondary analysis. The study data were deidentified. Participants were compensated with a nominal transport allowance of SGD $80 (US $59.14) for each of the clinic visits at weeks 0, 12, and 24.

## Results

### Participant Characteristics

A total of 25 participants were recruited and followed up over 24 weeks. The baseline demographic and clinical characteristics are summarized in Table 1. The study cohort had a mean age of 58.9 (SD 7.0) years, was 56% (n=14) male, and had a mean BMI of 29.0 (SD 3.6) kg/m^2^.

**Table 1.** Baseline demographic and clinical characteristics of 25 adults with type 2 diabetes enrolled in the GEMINI-T2D^a^ study, a 24-week pre-post intervention study conducted at the Singapore General Hospital from September 2020 to May 2022. All participants were on twice-daily premixed insulin and met inclusion criteria for HbA_1c_^b^ level (7.5%‐9.9%), BMI (≤40 kg/m²), and experience with self-monitoring of blood glucose.

Characteristics	Value (N=25)
Age (years), mean (SD)	58.9 (7.0)
Sex, n (%)	
Male	14 (56)
Female	11 (44)
Ethnicity, n (%)	
Chinese	13 (52)
Malay	4 (16)
Indian	6 (24)
Others	2 (8)
BMI (kg/m^2^), mean (SD)	29.0 (3.6)
HbA_1c_ level (%), mean (SD)	8.6 (0.7)
HbA_1c_ level (mmol/mol), mean (SD)	70.4 (7.7)
Fasting plasma glucose (mmol/L), mean (SD)	8.7 (2.0)
Duration of diabetes (years), mean (SD)	18.9 (6.7)
Duration of insulin use (years), mean (SD)	6.3 (3.5)
Total daily dose of insulin (units/day), mean (SD)	57.3 (24.4)
Total daily dose of insulin (units/kg/day), mean (SD)	0.73 (0.31)
Hypertension, n (%)	22 (88)
Hyperlipidemia, n (%)	23 (92)
Ischemic heart disease, n (%)	3 (12)
Stroke, n (%)	1 (4)
Retinopathy, n (%)	12 (48)
Neuropathy, n (%)	4 (16)
Nephropathy, n (%)	6 (24)
Peripheral vascular disease, n (%)	2 (8)

a GEMINI-T2D: Glucose Monitoring and Intervention in Insulin-treated Type 2 Diabetes Patients.

b HbA_1c_: hemoglobin A_1c_.

### HbA_1c_ Level and Other Glycemic Variables

**Figure 1.**
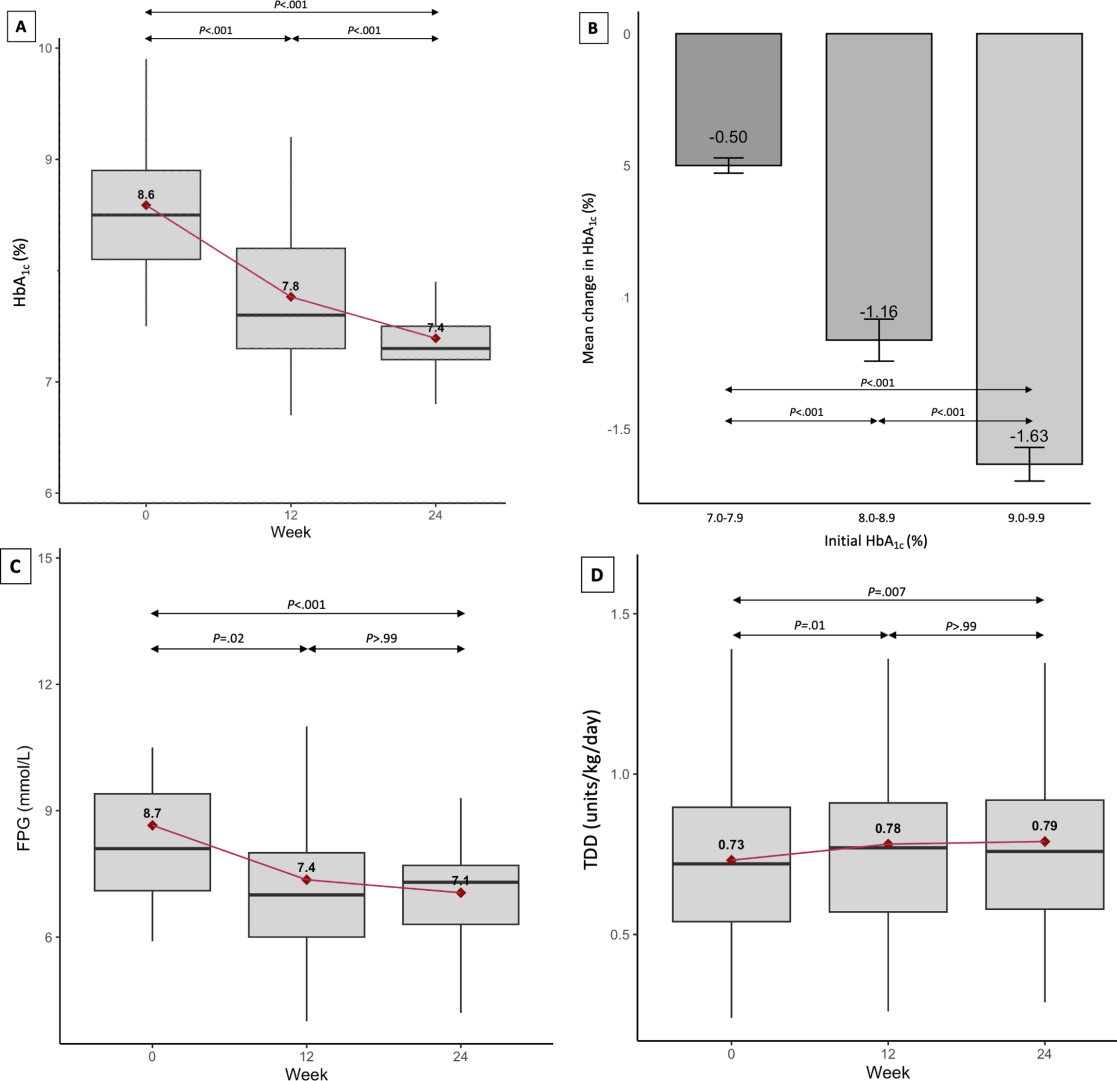
Trends in (A) hemoglobin A_1c_ (HbA_1c_) level, (B) mean change in HbA_1c_ level stratified by baseline HbA_1c_ level, (C) fasting plasma glucose (FPG), and (D) total daily dose (TDD) of insulin (units/kg/day) during the 24-week GEMINI-T2D study. The study assessed the impact of algorithm-guided insulin titration delivered via a web-based telehealth platform in 25 adults with insulin-treated type 2 diabetes at the Singapore General Hospital from September 2020 to May 2022. GEMINI-T2D: Glucose Monitoring and Intervention in Insulin-treated Type 2 Diabetes Patients.

Mean baseline HbA_1c_ level and FPG were 8.6% (SD 0.7%) and 8.7 (SD 2.0) mmol/L, respectively (Table 2). The mean baseline TDD of insulin was 57.3 (SD 24.4) units/day or 0.73 (SD 0.31) units/kg/day. All 25 participants experienced a reduction in HbA_1c_ level of ≥0.4% at 24 weeks. Mean HbA_1c_ level decreased by 1.2% over 24 weeks, from 8.6% (SD 0.7%) to 7.4% (SD 0.6%; *P*<.001; Figure 1). From weeks 0‐12, the mean HbA_1c_ level decreased by 0.8% (*P*<.001), and from weeks 13‐24, the mean HbA_1c_ level decreased by 0.4% (*P*<.001). Individuals with a higher baseline HbA_1c_ level experienced a greater absolute decrease in HbA_1c_ level over 24 weeks (*P*<.001; Figure 1).

Mean FPG also decreased by 1.6 mmol/L over 4 weeks, from 8.7 (SD 2.0) mmol/L to 7.1 (SD 1.4) mmol/L (*P*<.001; Figure 1). From weeks 0‐12, mean FPG decreased by 1.3 mmol/L (*P*=.02), and from weeks 13‐24, mean FPG decreased by 0.3 mmol/L (*P*>.99).

The mean TDD of insulin increased from 0.73 (SD 0.31) units/kg/day to 0.79 (SD 0.34) units/kg/day (*P*=.01; Figure 1). From weeks 0‐12, the mean TDD of insulin increased by 0.05 units/kg/day (*P*=.02), and from weeks 13‐24, the mean TDD of insulin increased by 0.01 units/kg/day (*P*>.99). BMI rose from 29.0 kg/m^2^ to 29.5 kg/m^2^ over 24 weeks (*P*=.03).

**Table 2.** Changes in key glycemic outcomes, insulin doses, and BMI from baseline to weeks 12 and 24 in the GEMINI-T2D^a^ study, which evaluated the efficacy of a web-based, algorithm-guided insulin titration system in adults with insulin-treated type 2 diabetes at the Singapore General Hospital from September 2020 to May 2022. *P* values indicate trends over the study period.

Variable	Baseline, mean (SD)	Week 12, mean (SD)	Week 24, mean (SD)	*P* value for trend
HbA_1c_^b^ level (%)	8.6 (0.7)	7.8 (0.6)	7.4 (0.6)	<.001
HbA_1c_ level (mmol/mol)	70.4 (7.7)	61.4 (7.0)	57.3 (6.7)	<.001
Fasting plasma glucose (mmol/L)	8.7 (2.0)	7.4 (1.9)	7.1 (1.4)	<.001
Total daily dose of insulin (units/day)	57.3 (24.4)	61.3 (25.7)	62.1 (25.4)	.01
Total daily dose of insulin (units/kg/day)	0.73 (0.31)	0.78 (0.33)	0.79 (0.34)	.007
BMI (kg/m^2^)	29.0 (3.6)	29.3 (3.6)	29.5 (3.6)	.04

a GEMINI-T2D: Glucose Monitoring and Intervention in Insulin-treated Type 2 Diabetes Patients.

b HbA_1c_: hemoglobin A_1c_.

### Hypoglycemia and Adherence to SMBG

At baseline, 44% (11/25) of participants reported at least 1 episode of hypoglycemia in the preceding month. During our study, 48% (12/25) of participants experienced at least 1 episode of hypoglycemia. In total, 24 episodes of hypoglycemia occurred in weeks 0‐12, while 72 episodes occurred in weeks 13‐24. The episodes of hypoglycemia were predominantly confined to level 1 (mild; blood glucose 3.0‐3.9 mmol/L), with 85 (89%) out of 96 episodes falling in this category (Table 3). There were no level-3 (severe) episodes of hypoglycemia reported during the study. Adherence to the prescribed SMBG frequency was 98.2% (165/168) for weeks 0‐12 and 96.5% (162/168) for weeks 12‐24.

**Table 3.** Incidence and severity of hypoglycemia episodes per participant during the 24-week GEMINI-T2D^a^ study. Episodes were categorized by level: mild (3.0‐3.9 mmol/L), moderate (<3.0 mmol/L), and severe (requiring external assistance). Data highlight an increase in mild hypoglycemia episodes from weeks 0‐12 to weeks 13‐24. The study was conducted in 25 adults with insulin-treated type 2 diabetes using a web-based telehealth platform at the Singapore General Hospital from September 2020 to May 2022.

Level of hypoglycemia	Incidence of hypoglycemia	
	Weeks 1‐12	Weeks 13‐24
1 (mild)	0.88	2.52
2 (moderate)	0.08	0.36
3 (severe)	0	0

a GEMINI-T2D: Glucose Monitoring and Intervention in Insulin-treated Type 2 Diabetes Patients.

## Discussion

The GEMINI-T2D study demonstrated that remote monitoring of SMBG data via a glucose management platform, coupled with algorithm-guided insulin titration, may improve glycemic control in patients with insulin-treated T2D. To the best of our knowledge, this is the first study of an integrated, mobile phone–based solution in an Asian cohort with insulin-treated diabetes. The degree of HbA_1c_ improvement in our study was comparable to studies on smartphone-based diabetes management platforms [15,18,22] and a larger trial on an automated insulin titration system [19].

Several key factors likely contributed to the improvement in glycemic control in our cohort. The ALRT system empowered individuals to participate in structured SMBG, through a platform that allowed for ease of upload of blood glucose readings directly from a glucometer. It also provided a convenient way to store, visualize, and communicate the data with their physicians, receiving timely feedback. The weekly review of data followed by insulin dose adjustments also tightened the feedback loop between patients and their physicians, enabling more prompt insulin dose adjustments than usually possible in a clinic setting. Physicians are also likely to have been empowered by the algorithm-guided insulin dose recommendations, which provide a framework to base their therapeutic decisions upon.

In addition, we observed that the improvement in HbA_1c_ level during the first half of the study persisted into the second half, demonstrating sustained benefits from the intervention. Interestingly, although the average increase in TDD of insulin was only 0.05 units/kg/day or 3 units/day for a 60-kg person, HbA_1c_ level decreased by 1.7%. This modest increase in insulin dose alone is unlikely to account for such a substantial improvement in glycemia. We speculate that the “outsized” glycemic improvement may be attributed to a more optimal redistribution of insulin doses facilitated by structured SMBG.

Another contributing factor to the improved glycemic control could be enhanced patient empowerment, which may have led to positive changes in overall health, diet, and lifestyle. This is supported by the high adherence to the prescribed SMBG regimen observed in our cohort, exceeding rates typically reported in the literature [23]. Enhanced patient engagement, consistently recognized as a critical factor in managing chronic diseases such as diabetes [24-26], was a central focus of our intervention. This engagement was fostered through patient education, twice-daily SMBG, and frequent review with feedback from physicians.

With regard to the safety of our intervention, while an increase in level-1 (mild) episodes of hypoglycemia occurred with tighter glycemic control, we found a low rate of level-2 (moderate) hypoglycemia, with no episodes of level-3 (severe) hypoglycemia. This safety signal was likely contributed by a few measures, such as patient education on hypoglycemia recognition and management at the time of enrollment, along with automated alerts with algorithm-guided insulin dosing suggestions to the treating physician via the ALRT app in event of hypoglycemia, prompting timely adjustment of insulin doses ahead of the scheduled weekly SMBG reviews. The safety profile aligns with a US-based randomized controlled trial [19] that showed similar hypoglycemia rates with automated insulin titration guidance compared to standard care. Nevertheless, dynamic adjustments to the algorithm to minimize hypoglycemia rates will be warranted in future implementation of this intervention in other populations.

A web-based platform with algorithm-guided insulin dose titration holds promise for closing the titration gap for people on insulin therapy, offering dynamic and frequent adjustments based on data, while assisting busy physicians in delivering necessary care safely and conveniently. Moving forward, it is also fathomable that artificial intelligence may allow for yet larger scales of SMBG data management and analysis, and provide more personalized insulin dosing recommendations that take into account factors beyond glycemic trend such as diet, lifestyle, and general health.

The primary limitation of our study is a small sample size, which reflects its design as a pilot study of an intervention. While the pre-post intervention design of our study suggests the effectiveness of our intervention compared to prior standard care, a longer study with a larger sample size would be warranted to evaluate the long-term effectiveness and scalability of such a web-based platform in diabetes care. Incorporating a comparison arm would further clarify the intervention’s performance against standard care and help identify the factors contributing to the improved glycemic control observed in our cohort. Additionally, our strict inclusion criteria may limit the generalizability of our findings to a broader population with diabetes, warranting further exploration. Finally, understanding physicians’ experiences with this intervention would provide valuable insights into the practical challenges and opportunities in its integration into routine clinical practice.

In conclusion, patients with insulin-treated T2D initiated on a web-based glucose monitoring system with algorithm-guided dosing recommendations showed significant improvement in glycemic control compared to baseline. High adherence rates underscore the feasibility of integrating algorithm-guided insulin titration into routine care. While hypoglycemia incidence rose slightly, episodes were predominantly mild, and no severe events occurred. This intervention shows promise for broader adoption in T2D management, particularly in resource-constrained settings.

## Supplementary material

10.2196/68914 Multimedia Appendix 1 Insulin dose titration algorithm.

## Abbreviations

FPG: fasting plasma glucose
GEMINI-T2D: Glucose Monitoring and Intervention in Insulin-treated Type 2 Diabetes Patients
HbA_1c_: hemoglobin A_1c_
SMBG: self-monitoring of blood glucose
T2D: type 2 diabetes
TDD: total daily dose
