# Supplementary material for: Web-Based, Algorithm-Guided Insulin Titration in Insulin-Treated Type 2 Diabetes: Pre-Post Intervention Study
Source: JMIR Form Res. 2025 Feb 7;9:e68914. doi: 10.2196/68914 (PMC11830485; doi:10.2196/68914)
Supplement: Multimedia Appendix 1 [file formative-v9-e68914-s001.docx]

**Multimedia Appendix 1: Insulin dose titration algorithm**

- Dose titration algorithm for twice daily premixed insulin
  - Capillary blood glucose (CBG) monitoring frequency:
    - At least twice a day and up to 4 times a day
    - Pre-breakfast and pre-dinner (optional: pre-lunch or bedtime)
    - Study participants will upload CBG data from their glucometer to the ALRT system every seven (7) days using the study app
- CBG targets
  - Pre-prandial CBG: 4 – 8 mmol/L
- Dose titration algorithm
  - Premixed insulin dose titration will be based on pre-breakfast and pre-dinner CBG readings
  - Hyperglycemia: increase corresponding dose by 10-15% or 1-2 units (whichever is lower)
    - E.g. Pre-breakfast dose: increase by 10-15% or 1-2 units if the lowest pre-dinner CBG on the preceding 7 nights was >8.0 mmol/L
    - E.g. Pre-dinner dose: increase by 10-15% or 1-2 units if the lowest pre-breakfast CBG on the preceding 7 mornings was >8.0 mmol/L
  - Hypoglycemia: decrease corresponding dose by 10-20% or 2-4 units whichever is higher
    - If sleeping hypoglycemia occurs, reduce pre-dinner dose
    - If daytime hypoglycemia occurs between meals (post-breakfast, pre-lunch, post-lunch, pre-dinner), reduce pre-breakfast dose.
    - For safety reason, all study participants will be counselled on hypoglycemia recognition and appropriate rescue measures.
    - All study participants will be advised to upload CBG data from their glucometer to the study App after an episode of hypoglycemia is confirmed via finger-prick testing.
